# A novel necroptosis-related long noncoding RNA model for predicting clinical features, immune characteristics, and therapeutic response in clear cell renal cell carcinoma

**DOI:** 10.3389/fimmu.2023.1230267

**Published:** 2023-08-02

**Authors:** Lei Zhang, Yongquan Chen, Weijing Hu, Bo Wu, Linfeng Ye, Dongwen Wang, Tao Bai

**Affiliations:** ^1^ Department of Urology, First Hospital of Shanxi Medical University, Taiyuan, China; ^2^ Department of the First Clinical Medical College, Shanxi Medical University, Taiyuan, China; ^3^ Department of Otorhinolaryngology-Head and Neck Surgery, Zhongnan Hospital of Wuhan University, Wuhan, China; ^4^ Cancer Hospital Shenzhen Hospital, Chinese Academy of Medical Sciences and Peking Union Medical College, Shenzhen, China; ^5^ Department of Pathology, First Hospital of Shanxi Medical University, Taiyuan, China

**Keywords:** necroptosis, lncRNA, ccRCC, prognosis, tumor microenvironment, immunotherapy, immune checkpoint inhibitors

## Abstract

**Background:**

Necroptosis is an immune-related cell death pathway involved in the regulation of the tumor microenvironment (TME). Here, we aimed to explore the role of necroptosis in clear cell renal cell carcinoma (ccRCC) and construct a necroptosis-related lncRNA (NRL) model to assess its potential association with clinical characteristics and immune status.

**Methods:**

Gene expression profiles and clinical data for ccRCC patients were obtained from the Cancer Genome Atlas (TCGA). Pearson’s correlation, univariate Cox, and least absolute shrinkage and selection operator analyses were used to develop an NRL model. Kaplan–Meier (K-M) and receiver operating characteristic (ROC) curve analyses were used to determine the prognostic value of the NRL model. The clinical information was used to assess the diagnostic value of the NRL model. The TME, immune function, immune cell infiltration, and immune checkpoints associated with the NRL model risk score were studied using the ESTIMATE, GSEA, ssGSEA, and CIBERSORT algorithms. The immunophenoscore (IPS) and half-maximal inhibitory concentration (IC50) were used to compare the efficacies of immunotherapy and chemotherapy based on the NRL model. Finally, *in vitro* assays were performed to confirm the biological roles of NRLs.

**Results:**

A total of 18 necroptosis-related genes and 285 NRLs in ccRCC were identified. A four-NRL model was constructed and showed good performance in the diagnosis and prognosis of ccRCC patients. The ESTIMATE scores, tumor mutation burden, and tumor stemness indices were significantly correlated with NRL model risk score. Immune functions such as chemokine receptors and immune receptor activity showed differences between different risk groups. The infiltration of immunosuppressive cells such as Tregs was higher in high-risk patients than in low-risk patients. High-risk patients were more sensitive to immunotherapy and some chemotherapy drugs, such as sunitinib and temsirolimus. Finally, the expression of NRLs included in the model was verified, and knocking down these NRLs in tumor cells affected cell proliferation, migration, and invasion.

**Conclusion:**

Necroptosis plays an important role in the progression of ccRCC. The NRL model we constructed can be used to predict the clinical characteristics and immune features of ccRCC patients.

## 1 Introduction

Clear cell renal cell carcinoma (ccRCC) is the most common renal cell carcinoma originating from tubular epithelial cells of the kidney (1). RCC is among the 10 most common cancers in both men and women. According to cancer statistics, there was an estimated 81, 800 new diagnoses of renal cancer and an estimated 14, 890 deaths from RCC in the United States in 2023. Globally, an estimated of 431, 288 people were newly diagnosed with renal cancer and an estimated 179, 368 deaths in 2020. Over the past 20 years, the global incidence of RCC has increased by approximately 2% per year (2). At the time of initial diagnosis, approximately 30% of patients with RCC has already metastasized, and in nearly 30% of patients with RCC, the cancer eventually metastasizes even after radical surgery (1). Despite the successes of newly targeted therapies and immune checkpoint blockades in improving clinical outcomes in metastatic RCC, the majority of metastatic RCC still ultimately lead to the death of patients (3, 4). Therefore, it is important to explore the underlying mechanisms of ccRCC and identify individualized biomarkers for different populations and more effective predictive targets.

Cell death is an important process in human development. Normal cell death can maintain the function and morphology of tissues and ensure the normal life activities of the human body. Necroptosis is a form of programmed cell death that is regulated by a series of molecular mechanisms (5–7). Unlike apoptosis, necroptosis is caspase-independent, has a strong immunoinflammatory response, and can induce immune system activation (8–11). Necroptosis is triggered by death receptors such as tumor necrosis factor receptor 1 and depends on the activation of receptor-interacting protein kinase 1 (RIPK1) and protein mixed lineage kinase domain-like (MLKL) (12). Necroptosis is closely related to the tumor immune microenvironment (13) and is a key process in tumorigenesis, cancer progression, and metastasis (14–17). However, because both tumor suppressing and promoting effects have been reported, the role of necroptosis in tumor development is still not fully understood (17, 18).

Long noncoding RNAs (lncRNAs) are RNA molecules that exceed 200 nucleotides in length and lack protein-coding potential (19). Increasing evidence indicates that lncRNAs play an important role in tumor development and prognosis (20). Xing C et al. demonstrated that lncRNA LUCAT1 can regulate tumor proliferation, invasion and migration through multiple mechanisms (21). Jiang T et al. found that targeting lncRNA DDIT4-AS1 can have an impact on chemotherapy drug sensitivity in breast cancer (22). These results suggest that lncRNAs may play an important role in the biological behavior and drug sensitivity of various tumors. However, the roles of necroptosis-related lncRNAs (NRLs) in ccRCC remain unclear. In this study, we characterized the expression of necroptosis-related genes in ccRCC and constructed an NRL model to assess their diagnostic and prognostic value, explore their impact on the immune microenvironment, and evaluate their potential for guiding clinical treatment decisions. This study can provide more precise treatment strategies and guide clinical management to improve the prognosis of ccRCC.

## 2 Materials and methods

### 2.1 Data acquisition and processing

Normalized gene expression data (in the format of TPM), somatic mutation, copy number variation data, and clinical information of ccRCC patients were downloaded from TCGA (https://portal.gdc.cancer.gov/). Gene expression data were collected from 537 ccRCC and 72 adjacent normal tissues. Clinical information included age, sex, clinical stage, grade, T stage, M stage, N stage, survival status, and survival time of 539 patients. These RNA-seq data were annotated using R software, and for different expression values of the same gene, we selected the mean value for analysis and screened the expression matrix for mRNA and lncRNA. The “limma” R package (23) was used to correct gene expression data.

### 2.2 Analysis of expression characteristics of key necroptosis genes

A total of 159 necroptosis-related genes were obtained from the KEGG database (24) (https://www.genome.jp/entry/hsa04217). An expression matrix of these 159 key necroptosis-related genes was extracted. The “clusterProfiler” R package was used for functional enrichment analysis. The STRING database (25) was used to identify interactions between the encoded proteins. The “limma” R package was designed to identify differentially expressed necroptosis-related genes in ccRCC. *P*< 0.05 was considered statistically significant. The “pheatmap” and “survival” R packages were used to visualize the expression profiles of key apoptosis-related genes. The “corrplot” R package used Spearman correlation analysis to identify potential correlations between genes. The “igraph” R package was used to draw co-expression maps. The “maftools” R package was used to visualize mutations in key necroptosis-related genes.

### 2.3 Screening of potential regulatory NRLs

The two-sample Wilcoxon rank-sum test and Spearman correlation analysis were used to screen differentially expressed NRLs (DE-NRLs). The threshold was set at |R| > 0.6 and *P<* 0.001. Cox regression analysis was used to screen for potential regulatory NRLs, and NRLs with *P<* 0.001 were used to construct the model.

### 2.4 Construction of a prognostic NRL model

To develop a prognostic model, 530 ccRCC patients with survival data were randomly assigned to training and test cohorts at a 1:1 ratio. The “glmnet” R package was used to perform a least absolute shrinkage and selection operator (LASSO) regression analysis in the training cohort to construct an NRL model risk score, which was calculated as follows: risk score = ∑Coef _lncRNA_ × Exp _lncRNA_, where Coef is the regression coefficient.

### 2.5 Evaluation of the diagnostic and prognostic performance of the NRL model

The patients were divided into low- and high-risk groups based on the median risk score. Principal component analysis (PCA) was used to analyze the clustering ability of the NRL model risk score. The log-rank test was used to compare overall survival (OS) between the two groups. The stability of the model was tested in the test cohort and the entire TCGA cohort. Univariate and multivariate Cox regression analyses were used to assess whether the risk score could be used as an independent prognostic factor. In addition, we analyzed the association of the NRL model risk score with other clinical parameters, such as gender, age, tumor grade, and stage, to assess the diagnostic predictive power of the model. The “ROC” R package was used to construct receiver operating characteristic (ROC) curves, and the area under the curve (AUC) was used to assess the specificity and sensitivity of the model.

### 2.6 Construction of a nomogram to predict survival

We constructed a prognostic nomogram combining the risk score and prognostic parameters to predict the 1-, 3-, and 5-year survival rates in patients with ccRCC. We assessed the reliability and accuracy of the nomogram using calibration curves, ROC curves, and decision curve analysis (DCA). The “rms” R package was used to plot the nomogram.

### 2.7 The relationship between the NRL model risk score and TME

TME scores for ccRCC samples were calculated using the ESTIMATE algorithm (26) to assess differences in the TME between the two groups. The correlation between TMB and NRL scores was analyzed using the “maftools” R package. Differences in risk scores across immunophenotypes (27) were analyzed using the “limma” R package. We also performed a correlation analysis between the risk score and TSI data from Tathiane M. Malta et al. (28), where RNAss is an index calculated based on expression data and DNAss is an index calculated based on gene methylation data.

### 2.8 The relationship between the NRL model risk score and immune cell infiltration

GSEA version 4.1.0 (http://www.broad.mit.edu/gsea/) was used to analyze the pathways associated with differentially expressed genes between the high- and low-risk groups. Single-sample gene set enrichment analysis (ssGSEA) was performed using the “GSVA” R package to assess intergroup differences in immune functions and cell subsets. The CIBERSORT, CIBERSORT abs mode (29), XCELL (30), TIMER (31), QUANTISEQ (32), MCPCOUNTER (33), and EPIC (34) algorithms were used to calculate the level of immune cell infiltration, and Spearman correlation analysis was used to detect the correlation between the risk score and level of immune cell infiltration.

### 2.9 The relationship between the NRL model risk score and drug sensitivity

The “limma” R package was used to analyze differences in the levels of immune checkpoint and immunosuppressive treatment-critical genes between the high- and low-risk groups. Tumor immunophenoscores (IPSs) of patients with KIRC were obtained using the TCIA database (https://tcia.at/home), and the correlation between IPS and the NRL model risk score was analyzed to predict sensitivity to immunotherapy. The “pRRophetic” R package (35) was used to predict half-maximal inhibitory concentrations (IC50) of targeted drugs commonly used clinically for ccRCC. The half-maximal inhibitory concentrations were compared between the high- and low-risk groups using the two-sample Wilcoxon rank-sum test.

### 2.10 Tissue sample and quantitative polymerase chain reaction analysis

Twenty pairs of ccRCC and matched normal tissues (confirmed by the pathology department of our center) were collected from patients who underwent radical nephrectomy in the Department of Urology of the First Hospital of Shanxi Medical University between June 2021 and December 2021. The study was approved by the Ethics Committee of the First Hospital of Shanxi Medical University (Approval No. 2021K034), and all patients provided written informed consent. The tissue was excised and immediately transferred to RNA protection solution. Total RNA was extracted from the tissues and cell lines using the *TransZol* Up Reagent (ET111-01, TransGen Biotech, China). RNA concentration and purity were determined using a spectrometer (Droplight304, Azanno Biotech, Sweden). Uni qPCR Kit Reagent (AUQ01, TransGen Biotech) was used to reverse transcribe the RNA template and assess the levels of four lncRNAs (AC016773.2, AC024060.2, AC026401.3, and EMX2OS); GAPDH was used as the internal reference gene. Quantitative polymerase chain reaction was performed using a real-time fluorescence quantitative PCR instrument (ABI 7500 Fast, USA). Primers designed using NCBI Primer-BLAST tool (https://www.ncbi.nlm.nih.gov/tools/primer-blast/) are shown in **Table 1**.

**Table 1:** Primer sequences.

Gene	Forward primer	Reverse primer
AC016773.2	GAAAGCGCATGGGCGCAG	CCCCTTCCGTTGGACTCAATC
AC024060.2	TCCCGAATGTGTGTTGCCTT	TTGCCATACAAGGTGGCTGG
AC026401.3	GAATTACGCTGCGATGGTGG	AAGCCTCTTTGACCAGAAGCC
EMX2OS	ATCCCTCCTCAGAACCCCTC	AAACATGCAAAGACCGTGCC
GAPDH	GTCCACCACCCTGTTGCTGTA	ACCCACTCCTCCACCTTTGA

### 2.11 Cell culture and RNA interference

The human normal renal epithelial cell line 293T, ccRCC cell lines 786-O, 769-P and ACHN were obtained from the Shanghai Cell Bank of the China Academy of Sciences. The cells were cultured in corresponding medium (Procell, China) containing 10% fetal bovine serum (10099-141, Gibco, USA) at 5% CO_2_ and 37°C. Small interfering (siRNA) was purchased from Hanbio (Shanghai, China); detailed information can be found in the **Supplementary Material**. For transfection, 786-O and 769-P cells (1 × 10^5^ cells/well) were grown in 6-well plates. When the cells reached 70% confluence, siRNA was transfected into the cells using RNAFit Reagent (HB-RF-1000, Hanbio, China) to achieve gene silencing. Quantitative polymerase chain reaction was used to assess the knockdown efficiency. Transfected cells were used for further experiments.

### 2.12 Detection of cell proliferation

The Cell Counting Kit-8 (CCK8) assay was used to detect the proliferative ability of ccRCC cells under different conditions. Twenty-four hours after transfection, transfected cells were seeded in 96-well plates in five replicates at 100 µL per well in RPMI-1640 medium containing 10% fetal bovine serum (approximately 5000 cells per well). The cells were incubated at 5% CO_2_ and 37°C for 0, 24, 48, 72, or 96 h, at which point 10 µL of Cell Counting Kit-8 reagent (HY-K0301, MCE, USA) was added to each well. Cells were incubated at 37°C for 1 h, and the absorbance at 450 nm was measured using a microplate reader (VICTOR Nivo, PerkinElmer, USA).

### 2.13 Detection of cell migration and invasion ability

Wound healing and Transwell assays were used to assess the migration and invasion abilities of ccRCC cells, respectively. Wound healing assays were performed in 6-well plates. Twenty-four hours after transfection, scratches were made with a 20-µL tip, the cells were rinsed twice with PBS, and the scratched area was photographed immediately after addition of serum-free RPMI-1640 medium (0 h). Cells were then incubated for 16 h at 5% CO_2_ and 37°C, and selected areas were photographed using an inverted microscope (DMIL, Leica, Germany). To assess invasion, 100 µL of prepared Matrigel (082704, ABW, China) was spread evenly in the upper chamber of a Transwell chamber (BD-353097, Falcon, USA) and incubated at 37°C for 3 h. After the Matrigel had set, 8 × 10^4^ transfected cells in 200 µL of serum-free RPMI-1640 medium were seeded in the upper chamber of the Transwell. The lower chamber contained 500 µL RPMI-1640 medium with 20% fetal bovine serum. After 24 h at 5% CO_2_ and 37°C, the cells that had reached the lower chamber were fixed with 0.4% paraformaldehyde for 15 min and stained with 0.1% crystal violet for 10 min. The membrane was washed with PBS, and fields were randomly selected and photographed using an inverted microscope.

### 2.14 Statistical analysis

All statistical analyses were performed using R software (version 4.1.3) and GraphPad Prism (Version 8.3.0). Two-sample Wilcoxon rank-sum test was used to analyze the differentially expression of necroptosis-related mRNAs and lncRNAs. Cox regression analysis was used to identify the prognostic value of NRLs. The log-rank test was used to analyze the survival rates of patients in different risk groups. Chi-square test was used to assess the association between NRL model risk score and clinical parameters. T-test was used to compare the difference between two groups. ANOVA was used to compare differences between three or more groups. *P*< 0.05 was considered statistically significant. (*: *P*< 0.05, **: *P*< 0.01, and ***: *P*< 0.001).

## 3 Results

### 3.1 Expression characteristics of necroptosis-related genes in ccRCC

**Figure 1:**
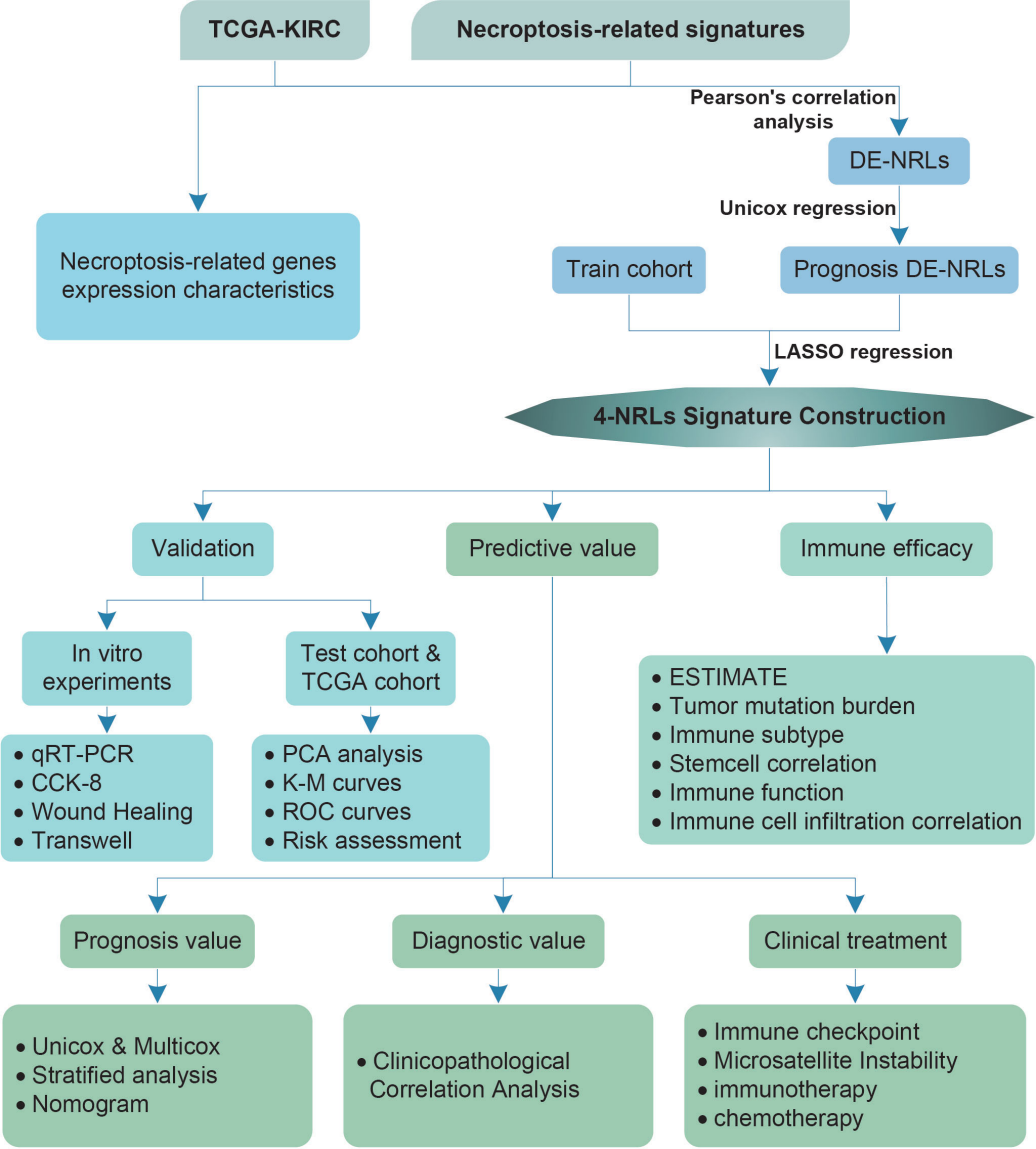
Flowchart of the study analysis.

**Figure 2:**
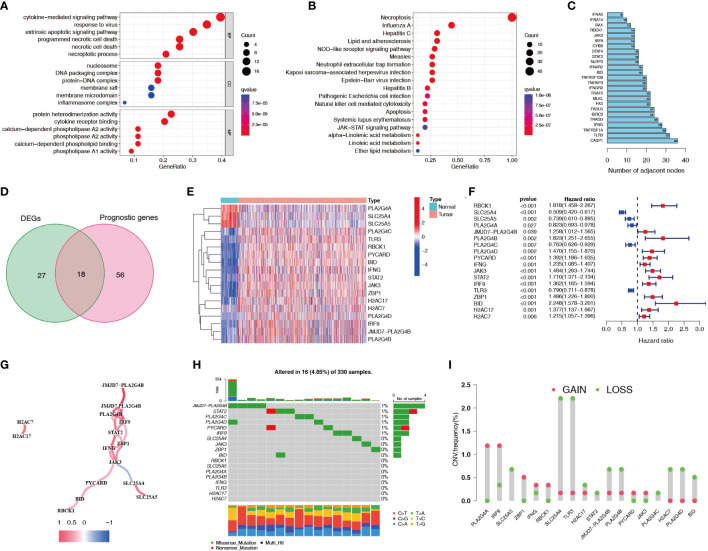
Expression characteristics of necroptosis-related genes for ccRCC patients in the TCGA dataset. **(A, B)** GO and KEGG functional enrichment analyses of genes related to necroptosis. **(C)** Protein interaction analysis of necroptosis-related genes. Numbers represent the number of adjacent nodes. **(D)** Venn diagram of necroptosis-related differentially expressed genes and prognostic genes. **(E, F)** Differential expression heatmap and prognostic forest plot of key necroptosis-related genes. **(G)** Co-expression analysis of key necroptosis-related genes. Red indicates a positive correlation and blue indicates a negative correlation. **(H)** SNPs in key necroptosis-related genes. Different colors represent different types of mutations. The numbers on the left side of the upper bar graph represent tumor mutation burden, and the percentages on the right represent mutation frequency. **(I)** Frequency of copy number variations (amplifications and deletions) in key necroptosis-related genes. TCGA, The Cancer Genome Atlas; GO, Gene Ontology; KEGG, Kyoto Encyclopedia of Genes and Genomes; SNP, single nucleotide polymorphism.

A flowchart of this study is shown in **Figure 1**. 45 differentially expressed necroptosis-related genes were screened. GO and KEGG analyses showed that differentially expressed necroptosis-related genes in ccRCC patients were enriched in necroptosis, NOD-like receptor, and JAK signaling pathways (**Figures 2A, B**). Protein interaction analysis showed that the necroptosis-related proteins CASP1, TLR3, and TNFRSF1A play an important role in ccRCC (**Figure 2C**). Combined with prognostic correlates and differentially expressed necroptosis-related genes, 18 differentially expressed necroptosis-related genes that were correlated with prognosis were found to play a critical role (**Figures 2D–F**). The co-expression analysis showed a positive correlation between the expression of most necroptosis-related genes and a negative correlation between the expression of JAK3 and SLC25A4 (**Figure 2G**). Single nucleotide polymorphism analysis showed that 16 patients had mutations in key necroptosis genes, most of which were deletion mutations, and the mutation frequency of JMJD7-PLA2G4B was relatively high (up to 1.2%) (**Figure 2H**). Copy number variation analysis showed different degrees of copy number variation in necroptosis-related genes, most of which were copy number deletions. The frequencies of copy number deletions in SLC25A4 and TLR3 were relatively high (up to 2.2%), and the frequencies of copy number amplification in PLA2G4A and IRF9 were also relatively high (less than 1.5%) (**Figure 2I**). These results suggest that the expression of these key necroptosis genes is altered in various forms and may play an important role in the genesis and development of ccRCC.

### 3.2 NRL screening and model construction

**Figure 3:**
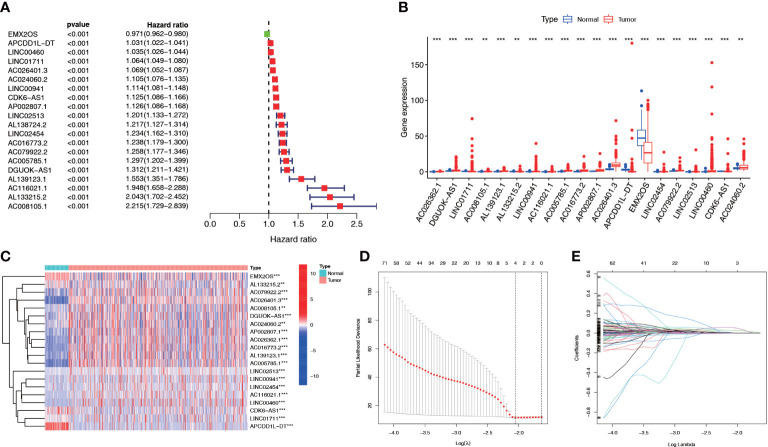
NRL screening and model construction. **(A-C)** Single factor Cox regression and differential analyses of some necroptosis lncRNAs. **(D, E)** LASSO regression analysis of the NRL model. **, *P*< 0.01; ***, *P*< 0.001. NRL: necroptosis-related long noncoding RNA; lncRNA: long noncoding RNA; LASSO, least absolute shrinkage and selection operator.

We screened 1845 DE-NRLs using differential analysis (**Supplementary Table 1**). Univariate Cox regression analysis revealed that 285 DE-NRLs were significantly associated with ccRCC prognosis (**Supplementary Table 2**). Twenty DE-NRLs were randomly selected from the results, and prognostic forest plot, differential expression boxplot, and differential expression heatmap were shown in **Figures 3A–C**. The data from 530 patients with ccRCC from TCGA were divided into two groups: 264 patients were included in the training cohort and 266 patients were included in the test cohort. The clinical parameters of the groups are shown in **Table 2** (more detailed patient information can be found in **Supplementary Table 3**). We performed LASSO regression analysis on the 285 prognostic DE-NRLs in the training cohort (**Figures 3D, E**). Finally, a model containing four hub NRLs was established (**Supplementary Table 4**). The NRL score was calculated as follows:

**Table 2:** Clinical characteristics of KIRC patients involved in the study.

Characteristic	Training cohort (N=264)	Testing cohort (N=266)	TCGA cohort (N=530)
Age, n (%)			
<=65	169 (15.9%)	179 (16.9%)	348 (32.8%)
>65	97 (9.2%)	85 (8%)	182 (17.2%)
Gender, n (%)			
FEMALE	92 (8.7%)	94 (8.9%)	186 (17.5%)
MALE	174 (16.4%)	170 (16%)	344 (32.5%)
Grade, n (%)			
G1-2	117 (11%)	124 (11.7%)	241 (22.7%)
G3-4	145 (13.7%)	136 (12.8%)	281 (26.5%)
Unknown	4 (0.4%)	4 (0.4%)	8 (0.8%)
Stage, n (%)			
Stage I-II	158 (14.9%)	164 (15.5%)	322 (30.4%)
Stage III-IV	107 (10.1%)	98 (9.2%)	205 (19.3%)
Unknown	1 (0.1%)	2 (0.2%)	3 (0.3%)
T stage, n (%)			
T1-2	170 (16%)	170 (16%)	340 (32.1%)
T3-4	96 (9.1%)	94 (8.9%)	190 (17.9%)
M stage, n (%)			
M0	210 (19.8%)	210 (19.8%)	420 (39.6%)
M1	38 (3.6%)	40 (3.8%)	78 (7.4%)
Unknown	18 (1.7%)	14 (1.3%)	32 (3%)
N stage, n (%)			
N0	128 (12.1%)	111 (10.5%)	239 (22.5%)
N1	5 (0.5%)	11 (1%)	16 (1.5%)
Unknown	133 (12.5%)	142 (13.4%)	275 (25.9%)


risk score=AC016773.2×0.054+AC026401.3×0.027+EMX2OS×(−0.002)+AC024060.2×0.001

### 3.3 Evaluation of the prognostic value of the NRL model

**Figure 4:**
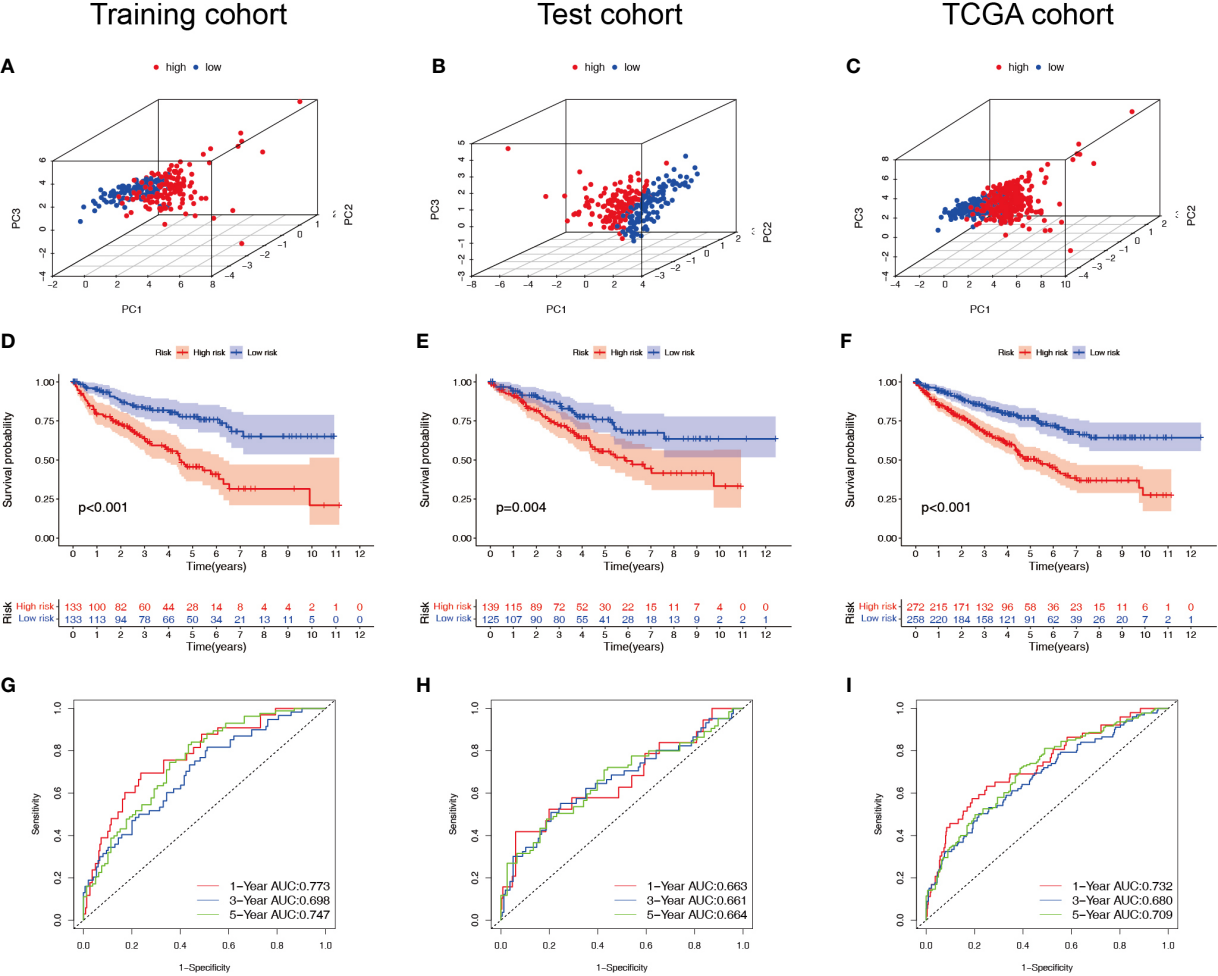
Survival analysis of the NRL model. **(A–C)** PCA plots of high- and low-risk patients in the training, test, and TCGA cohorts. **(D–F)** Overall survival of high- and low-risk patients in the training, test, and TCGA cohorts. **(G–I)** ROC curves for high- and low-risk patients in the training, test, and TCGA cohorts. NRL, necroptosis-related long noncoding RNA; PCA, principal component analysis; TCGA, The Cancer Genome Atlas; ROC, receiver operating characteristic.

**Figure 5:**
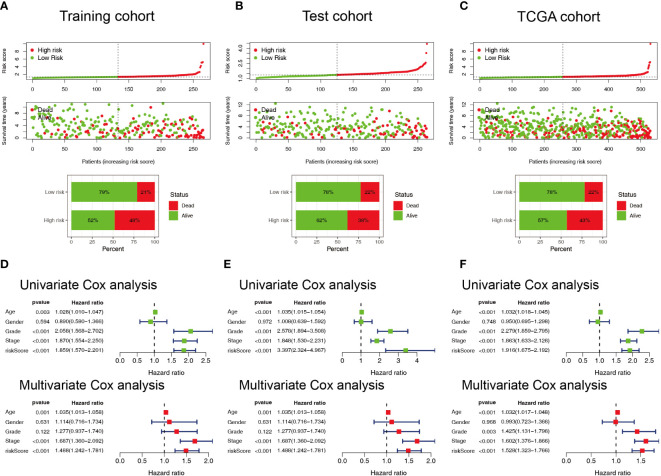
Evaluation of the predictive ability of the NRL model. **(A–C)** Scatter plot of the risk score and survival status in the training, test, and TCGA cohorts. **(D–F)** Forest plots of univariate and multivariate Cox analyses results for the training, test, and TCGA cohorts. NRL, necroptosis-related long noncoding RNA; TCGA, The Cancer Genome Atlas.

**Figure 6:**
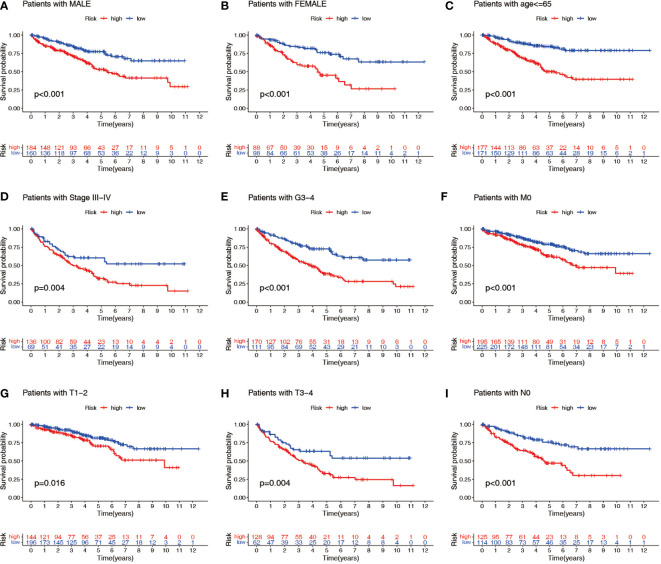
Stratified prognostic power assessment. K-M survival analysis between patients in the high- and low-risk groups in different clinical groups. Gender **(A, B)**, age **(C)**, stage **(D)**, grade **(E)**, M stage **(F)**, T stage **(G, H)** and N stage **(I)**. K-M, Kaplan–Meier; M, metastasis; T, tumor; N, node.

Patients with ccRCC were divided into low- and high-risk groups based on the median risk score. PCA showed that the risk score could cluster ccRCC patients (**Figures 4A–C**). Kaplan–Meier survival analysis showed that patients in the low-risk group had a better clinical prognosis than those in the high-risk group (**Figures 4D–F**; *P*< 0.001 for the training cohort, *P* = 0.004 for the test cohort, and *P*< 0.001 for the entire TCGA cohort). ROC curve analysis showed that the risk score had good predictive performance for OS in ccRCC patients (**Figures 4G–I**). The scatter plot analysis of the risk score and survival status showed that the survival status of patients with a high risk score was worse than that of patients with a low risk score, and their mortality was higher (**Figures 5A–C**). Univariate and multivariate Cox analyses showed that the NRL risk score could be used as an independent prognostic factor for ccRCC (**Figures 5D–F**). These conclusions were verified in both the test cohort and the entire TCGA cohort (**Supplementary Table 5**). In addition, stratified prognostic analysis showed that there was still a significant difference in prognosis between high- and low-risk patients among different clinical subtypes (**Figures 6A–I**). However, although the prognosis of patients in the high-risk group was worse in patients with low-grade tumors, low-stage tumors, N1 stage, and M1 stage, these differences were not significant (**Figure S1**).

### 3.4 Evaluation of the diagnostic value of the NRL model

**Figure 7:**
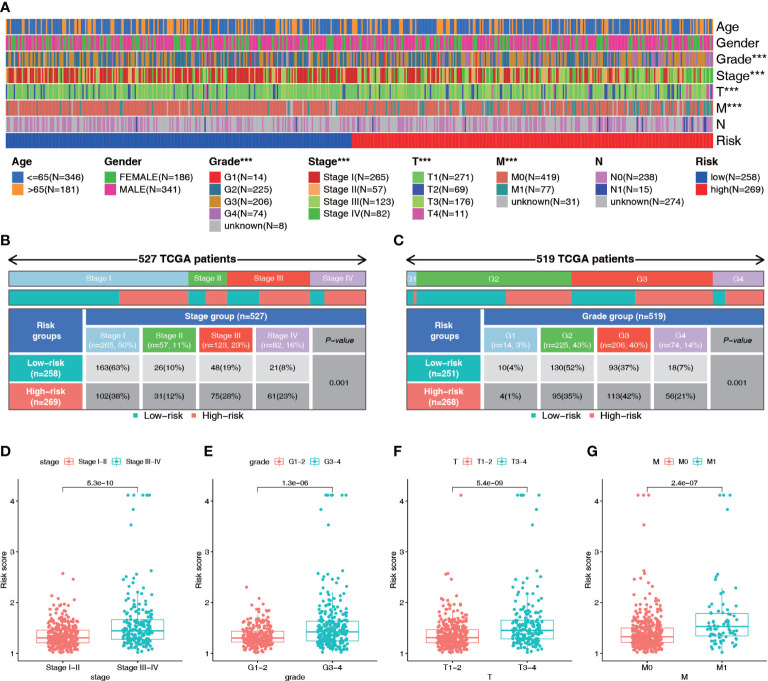
Diagnostic value of the NRL model. **(A–C)** Correlation analysis of risk score and clinical parameters (Stage and grade). **(D–G)** Differences in risk scores among patients with different clinical traits (Stage, grade, T stage and M stage). ***, *P*< 0.001. NRL, necroptosis-related long noncoding RNA.

Patients in the high-risk group generally had a higher tumor stage, grade, tumor volume, and distant metastasis risk than patients in the low-risk group (**Figures 7A–C**; **S2A–E**). Therefore, we analyzed the differences in the risk scores among patients with different clinical parameters. The results showed that there were significant differences in risk scores among patients with different stages, grades, and T, N, and M stages but no significant differences in risk scores based on age or gender (**Figures 7D–G**; **S2F–H**). Patients with higher grade and stage had higher risk scores. These results suggest that the NRL risk score may serve as a key indicator for clinical prediction in ccRCC patients.

### 3.5 Construction and evaluation of a prognostic nomogram

**Figure 8:**
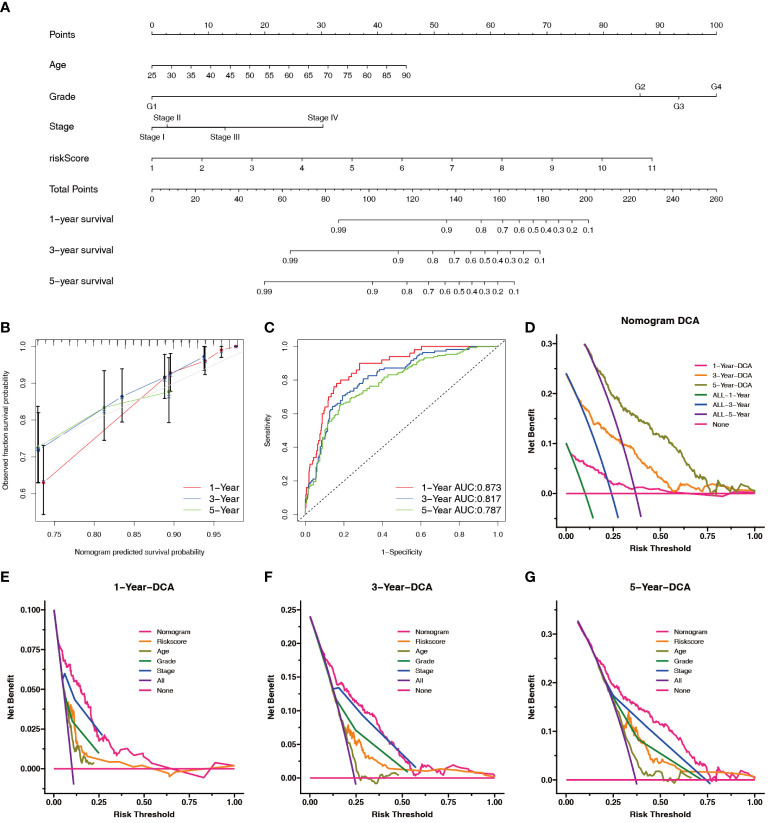
Construction and evaluation of the nomogram. **(A)** Prognostic nomograms were constructed based on the NRL model and clinical traits to predict the 1-, 3-, and 5-year OS in patients with renal cell carcinoma. **(B)** The 1-, 3-, and 5-year nomogram calibration curves. The 45-degree line represents the ideal prediction. **(C)** ROC curve analysis of the nomogram. **(D–G)** DCA showed clinical benefit at 1, 3, and 5 years. NRL, necroptosis-related long noncoding RNA; OS, overall survival; ROC, receiver operating characteristic; DCA, decision curve analysis.

To predict survival in patients with ccRCC more accurately, we created a clinically applicable prediction tool. Nomograms were constructed to predict the 1-, 3-, and 5-year survival probabilities by incorporating clinical parameters associated with patient prognosis (**Figure 8A**). The calibration curve and ROC curve analysis indicated excellent prognostic predictive abilities of the nomogram for the 1-, 3-, and 5-year overall survivals (AUC = 0.873, 0.817, and 0.787, respectively) (**Figures 8B, C**). DCA showed that the nomogram had a good net benefit and wide threshold probability range for predicting the 1-, 3-, and 5- overall survival rates. In addition, the nomogram had a better net clinical benefit than the NRL model (**Figures 8D–G**).

### 3.6 The relationship between the NRL model risk score and TME

**Figure 9:**
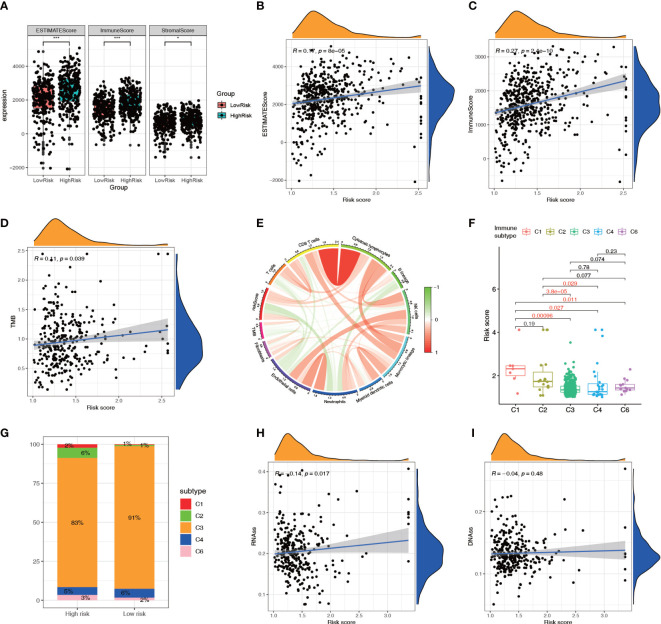
Relationship between the NRL model risk score and tumor microenvironment. **(A–C)** Relationship between the ESTIMATE score and risk score. **(D, E)** Correlation analysis of TMB with the risk score and immune cell infiltration. **(F, G)** Correlation analysis between immune subtypes and the risk score. **(H, I)** Correlation analysis between the tumor stemness index and risk score. *, *P*< 0.05; ***, *P*< 0.001. NRL, necroptosis-related long noncoding RNA; ESTIMATE, Estimation of STromal and Immune cells in MAlignant Tumor tissues using Expression data; TMB, tumor mutation burden.

The ESTIMATE analysis showed that the high-risk group had significantly higher ESTIMATE score and immune score than the low-risk group, and the ESTIMATE score and immune score were significantly positively correlated with the risk score (**Figures 9A–C**). The TMB correlation analysis showed that TMB was positively correlated with the risk score (**Figure 9D**), suggesting that the high-risk group had a higher TMB. The MCPCOUNTER analysis indicated that there was a negative correlation between the TMB and the levels of immune cells (**Figure 9E**). The immune subtype correlation analysis showed that the risk score of the C1 (Wound Healing) genotype group was relatively high, the score of the C3 (Inflammatory), C4 (Lymphocyte-Depleted) genotype group was relatively low (**Figure 9F**). The Sankey diagram showed higher proportions of high-risk patients in the C1 (Wound Healing), C2 (IFN-gamma Dominant), and C6 (TGF-beta Dominant) genotype groups (**Figure 9G**). Correlation analysis of the tumor stemness index showed that the risk score was significantly and positively correlated with the RNA stem cell index but not the DNA stem cell index (**Figures 9H, I**).

### 3.7 The relationship between the NRL model risk score and immune cell infiltration

**Figure 10:**
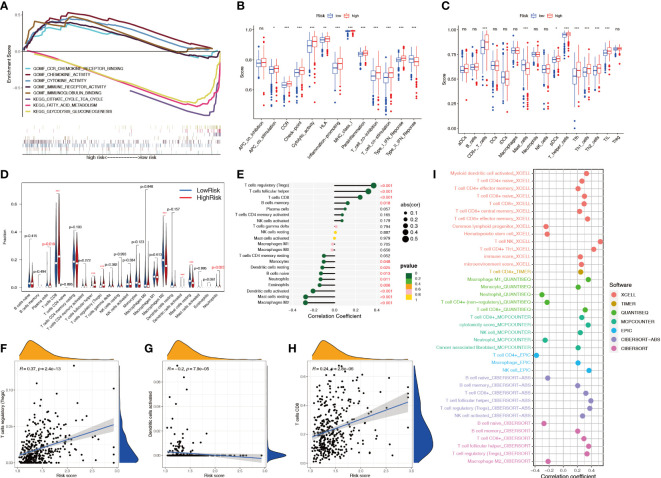
Relationship between the NRL model risk score and immune cell infiltration. **(A)** GSEA in different risk groups. **(B, C)** ssGSEA immune marker difference analysis in different risk groups. **(D)** Differences in CIBERSORT immune cell infiltration levels in different risk groups. **(E-H)** Correlation analysis between the risk score and the CIBERSORT immune cell infiltration level. **(I)** Correlation analysis between risk score and immune cell infiltration level using various algorithms. *, *P*< 0.05; **, *P*< 0.01; ***, *P*< 0.001; ns, no significance. NRL, necroptosis-related long noncoding RNA; GSEA, gene set enrichment analysis; ssGSEA, single-sample gene set enrichment analysis; CIBERSORT, Cell-type Identification By Estimating Relative Subsets Of RNA Transcripts.

GSEA showed that multiple metabolic pathways, such as fatty acid metabolism and citrate cycle tricarboxylic acid cycle, were activated in the low-risk group, suggesting that patients in the high-risk group had metabolic abnormalities, whereas chemokine and cytokine activities were activated in the high-risk group, suggesting that patients in the high-risk group had immune function-related abnormalities (**Figure 10A**). The ssGSEA showed that among 29 immune-related markers, there were 20 differences in immune function and immune cell markers between high-risk and low-risk patients, such as CCR and checkpoints (**Figures 10B, C**). CIBERSORT analysis was used to calculate the infiltration level of 22 immune cell types, and the results showed that eight immune cell types had different infiltration levels between high- and low-risk patients, including plasma cells, CD8+ T cells, regulatory T cells, dendritic cells, M2 macrophages, mast cells, and neutrophils (**Figure 10D**). Correlation analysis showed a significant positive correlation between Treg cells and the risk score and a significant negative correlation between M2 macrophages and the risk score (**Figure 10E**). The infiltration levels of Treg cells and CD8+ T cells increased with an increase in the risk score, whereas dendritic cells decreased with an increase in the risk score, indicating that patients in the high-risk group had higher levels of immunosuppression (**Figures 10F–H**; **S2**). The correlation between the immune cell infiltration level and risk score was studied by combining multiple algorithms. The results showed that CD8+ T cells and Treg cells were significantly positively correlated with the risk score, and activated B cells were negatively correlated (**Figure 10I**).

### 3.8 Evaluation of the clinical therapeutic predictive value of the NRL model

**Figure 11:**
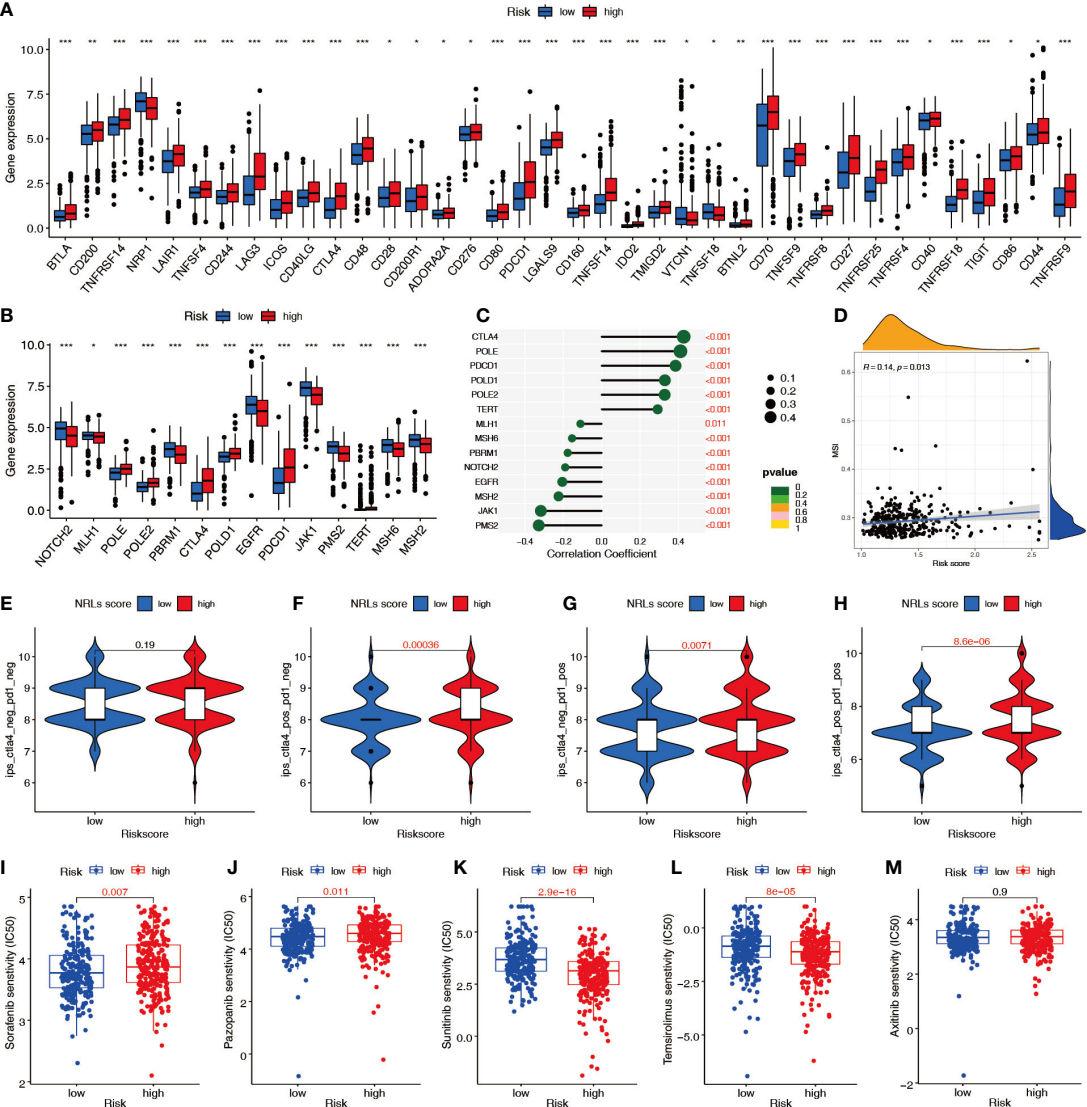
Predictive value of the NRL model risk score in clinical treatment. **(A)** Differences in immune checkpoint molecule expression between risk groups. **(B, C)** Relationship between the risk score and immunosuppressive treatment-related gene expression. **(D)** Correlation between the risk score and MSI. **(E–H)** IPSs were different in different risk groups. **(I–M)** Differences in sensitivity to targeted chemotherapy drugs among different risk groups (IC50). *, *P*< 0.05; **, *P*< 0.01; ***, *P*< 0.001. MSI, microsatellite instability; IPS, immunophenoscore; IC50, half-maximal inhibitory concentration.

Immune checkpoint analysis showed significant differences in 38 key immune checkpoints between patients in the high- and low-risk groups (**Figure 11A**), suggesting that the NRL model risk score can guide administration of immunotherapy. Analysis of the expression of PD-1 inhibitor efficacy-related genes revealed that the levels of the sensitive gene PBRM1, DNA mismatch repair genes MSH2, MSH6, MLH1, and PMS2, drug resistance gene JAK1, and outbreak gene EGFR were lower in the high-risk group than in the low-risk group, suggesting that high-risk patients are more likely to benefit from anti-PD-1 therapy (**Figure 11B**). The correlation analysis showed that the DNA mismatch repair genes MSH2, MSH6, MLH1, and PMS2 were significantly negatively correlated with the risk score (**Figure 11C**) and that MSI was positively correlated with the risk score (**Figure 11D**). To predict the response to immunotherapy, the IPS was calculated for patients receiving different treatments, including no treatment, anti-CTLA-4 monotherapy, anti-PD-1 monotherapy, and combination therapy. The IPSs showed that patients in the high-risk group had higher scores, indicating that immunotherapy has greater efficacy in high-risk patients and they are more suitable for immunotherapy (**Figures 11E–H**). The sensitivity difference analysis of commonly used targeted chemotherapeutic drugs for renal cancer showed that the low-risk group had higher sensitivity to sorafenib and pazopanib and the high-risk group had higher sensitivity to sunitinib and temsirolimus (**Figures 11I–M**).

### 3.9 Validation of biological functions of hub lncRNAs in ccRCC

**Figure 12:**
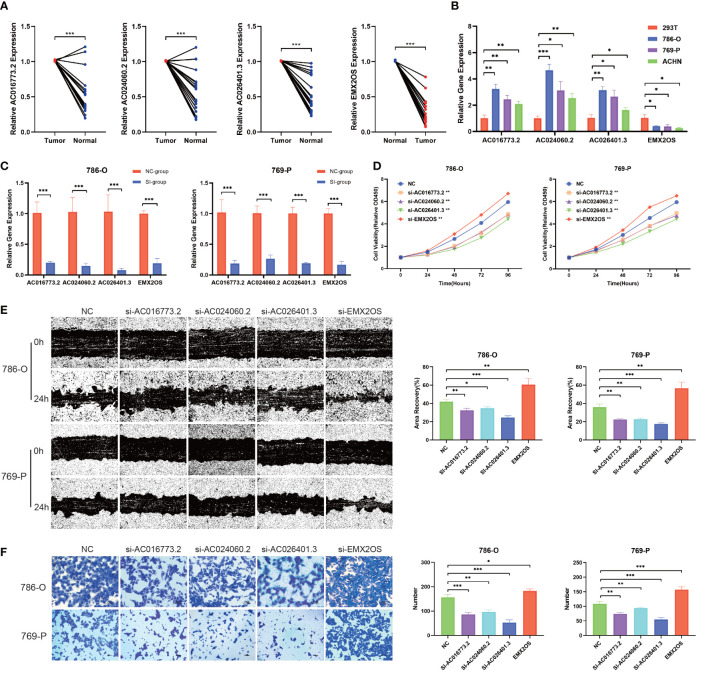
Validation of biological functions of hub lncRNAs in ccRCC. **(A)** Relative levels of the hub lncRNAs in 20 pairs of clinical tissue samples (N=20). **(B)** Relative levels of hub lncRNAs in ccRCC cells. **(C)** Evaluation of the siRNA transfection efficiency. **(D)** CCK8 assays were used to assess cell proliferation after transfection. **(E)** Wound healing assays were used to assess cell migration after transfection. **(F)** Transwell assays were used to assess cell invasion after transfection. *, *P*< 0.05; **, *P*< 0.01; ***, *P*< 0.001. lncRNA, long noncoding RNA; ccRCC, clear cell renal cell carcinoma; CCK8, Cell Counting Kit-8; 786-O, 769-P, two types of renal cancer cell lines.

To further examine the potential biological roles of the four hub lncRNAs in ccRCC, we first examined the expression of hub lncRNAs in ccRCC tissue samples and cells using qPCR. Compared with the paired adjacent tissues, the relative expression values of AC016773.2 were 1.01 ± 0.01 and 0.51 ± 0.29, the relative expression values of AC024060.2 were 1.01 ± 0.01 and 0.54 ± 0.31, the relative expression values of AC026401.3 were 1.01 ± 0.00 and 0.54 ± 0.25, and the relative expression values of EMX2OS were 0.29 ± 0.18 and 1.00 ± 0.01. The results showed that the relative levels of AC016773.2, AC024060.2, and AC026401.3 were significantly higher in ccRCC tissues and cells than in normal tissues and cells, and the relative levels of EMX2OS were significantly lower in ccRCC tissues and cells than in normal tissues and cells (*P<* 0.01, **Figures 12A, B**). Subsequently, siRNA was used to knock down the expression of hub lncRNAs in 786-O and 769-P cells. After knockdown, the levels of hub lncRNAs in transfected cells were significantly lower than those in non-transfected cells (*P*< 0.001, **Figure 12C**). The CCK-8 assay showed that the proliferation rates of 786-O and 769-P cells were lower after knockdown of AC016773.2, AC024060.2, and AC026401.3, whereas the proliferation rates of 786-O and 769-P cells were higher after EMX2OS knockdown (**Figure 12D**). The wound healing assay showed that the migration abilities of 786-O and 769-P cells were decreased after AC016773.2, AC024060.2, and AC026401.3 knockdown, whereas they were increased after EMX2OS knockdown (**Figure 12E**). The Transwell assay showed that the invasion abilities of 786-O and 769-P cells were decreased after AC016773.2, AC024060.2, and AC026401.3 knockdown and increased after EMX2OS knockdown (**Figure 12F**). More detailed experimental results are available in the **Supplementary Materials**.

## 4 Discussion

ccRCC is one of the most common urinary tract malignancies. Although there have been recent improvements in the treatment of ccRCC (36), early diagnosis, prognosis prediction, clinical diagnosis, and treatment guidance for ccRCC still require further study. Necroptosis is a new type of cell death regulation whose morphological characteristics are similar to those of necrosis, but the difference lies in whether it is regulated by signaling pathways (37). Recent studies have shown that necroptosis is closely associated with tumor progression (38–41), but little is known about the role of necroptosis in ccRCC. In this study, we characterized the expression of necroptosis-related genes, established an NRL model to predict OS, and evaluated its predictive power in terms of clinical outcomes, immunologic microenvironments, and response to immunotherapy.

We first investigated the expression and mutation profiles of 159 necroptosis-related genes in ccRCC patients in TCGA and found that 18 necroptosis-related genes play an important role in ccRCC. Some studies have shown that gene mutations play a guiding role in the occurrence of diseases such as cancer and the prognosis of cancer patients (42–45). Therefore, we explored the variation of 18 key necroptosis-related genes. The results of single nucleotide mutations showed that JMJD7-PLA2G4B and STAT2 were mutated in a relatively large number of people. The results of copy number variations showed that SLC25A4, TLR3, PLA2G4A and IRF9 were mutated at a relatively high frequency. These genes deserve more attention. Other studies have shown that these genes also play important roles in kidney cancer. One such gene, C3HC4-type zinc finger containing 1 (RBCK1), promotes p53 degradation through ubiquitination in renal cell carcinoma (46) and can be used as a key index for regulating the immune microenvironment (47). Another gene, interferon gamma (IFNG), is significantly associated with CD8+ T cell infiltration in renal cell carcinoma (48). We next performed LASSO regression analysis on selected key NRLs and constructed an NRL model containing four lncRNAs (AC016773.2, AC024060.2, AC026401.3, and EMX2OS). These lncRNAs are known to play important roles in a variety of cancers. For example, AC026401.3 acts on OCT1 to enhance drug resistance to sorafenib in hepatocellular carcinoma (49), and AC026401.3 regulates breast cancer progression by regulating CCNB1 (50). EMX2OS plays an important role in the prognosis of gastric cancer (51) and regulates proliferation and invasion of ovarian cancer through the miR-654-3p/AKT3/PD-L1 regulatory axis (52).

We divided patients with ccRCC into low- and high-risk groups according to the NRL model risk score. PCA showed that the patients could be divided into two clusters using the risk score. Kaplan–Meier survival analysis showed that the OS of patients in the high-risk group was shorter than that of patients in the low-risk group, and the scatter plot of survival status showed that a higher risk score was associated with worse survival. Univariate and multivariate Cox analyses suggested that the NRL model risk score was an independent prognostic risk factor. Survival analysis of patients with high and low risk in different clinical subgroups suggested that patients in the high-risk group had a poor prognosis. These results were verified in the training, test, and entire TCGA cohorts, confirming that the NRL model has prognostic value. In addition, we found that the risk score was correlated with clinical traits, suggesting that the NRL model can be used as a diagnostic and predictive indicator. We then constructed a nomogram that included the risk score to more accurately predict the 1-, 3-, and 5-year survival rates in patients with ccRCC. Calibration and ROC curves suggested that the nomogram was highly accurate, and DCA suggested that it had high clinical applicability.

The TME is closely related to tumor occurrence, growth, and metastasis (53). ESTIMATE analysis showed higher immune scores and total scores in high-risk patients, suggesting dysregulation of the TME and abnormal aggregation of immune cells in high-risk patients. TMB analysis indicated a higher TMB in high-risk patients, which may indicate that different subgroups of patients respond differently to immunotherapy (54). We identified a correlation between the risk score and different immune subtypes. Tumor stem cell correlation analysis showed that patients in the high-risk group had a higher stem cell index than patients in the low-risk group, which suggested that tumors in the high-risk group might have higher differentiation ability. GSEA indicated abnormalities in immune and metabolic signaling in the high-risk group, including in chemokine receptor activity, cytokines, and lipid metabolism. These results suggest that there are different degrees of immune dysfunction in the high- and low-risk groups. The ssGSEA indicated many abnormal immune markers, such as immune checkpoints and other key immune indicators. We then used the XCELL, TIMER, QUANTISEQ, EPIC, MCPCOUNTER, CIBERSORT, and CIBERSORT-ABS algorithms to compare the immune cell content in different risk score groups and found that the risk score was significantly positively correlated with Treg and CD8+ T cells and negatively correlated with dendritic cell activation. Treg cells have significant immunosuppressive effects, and dendritic cells can initiate a specific immune response, suggesting a certain degree of immunosuppression in high-risk groups. In addition, the high-risk group had a higher level of CD8+ T cell infiltration than the low-risk group, and current studies have found that high infiltration of CD8+ T cells in tumors indicates a state of dysfunction (55).

Immunotherapy plays an important role in the clinical treatment of ccRCC (56). At present, commonly used immunotherapeutic regimens include anti-PD-1 monoclonal antibodies, anti-CTLA-4 monoclonal antibodies, and combined immunotherapy. Therefore, we performed correlation analysis between immune checkpoint molecules and the NRL model risk score and found that the levels of most immune checkpoint genes, including CTLA-4, CD28, TNFSF18, CD27, and CD86, were significantly higher in high-risk patients than in low-risk patients, whereas the levels of NRP1 were lower in high-risk patients. These results suggest that these genes could be used as therapeutic targets for ccRCC. We then analyzed the relationship between the expression of genes that affect immunotherapy and the NRL model risk score and found significant anomalies. Previous studies have shown that patients with PBRM1 mutations are more sensitive to anti-PD-1 therapy, resulting in significantly improved treatment effects (57). In this study, patients in the high-risk group had lower PBRM1 expression, suggesting that the high-risk group may be more sensitive to anti-PD-1 therapy. Additionally, DNA mismatch repair genes play an important role in immunosuppressive drug therapy (58). DNA mismatch repair genes were significantly downregulated in high-risk patients in this study, suggesting that DNA repair is impaired in high-risk patients. Further, EGFR amplification can cause anti-PD-1 treatment flares (59). Our results showed that patients in the high-risk group had low EGFR expression, indicating that anti-PD-1 treatment may be safer in the high-risk group. The IPS indicated that patients in the high-risk group had better sensitivity to immunotherapeutic drugs and were more suitable for immunotherapy compared to patients in the low-risk group. Chemotherapy is an effective treatment for a variety of cancers, which has received extensive attention (60, 61). It is also widely used in renal cancer. Chemotherapy is an effective treatment for advanced renal cancer. We also evaluated sensitivity to common targeted chemotherapy drugs for ccRCC based on the NRL model risk score. Patients in the low-risk group were more sensitive to sorafenib and pazopanib, whereas patients in the high-risk group were more sensitive to sunitinib and temsirolimus. These results suggest that our NRL model risk score can be used to predict the response of patients to immunotherapy and chemotherapy and provide a direction for clinical treatment strategies. Finally, we experimentally verified the biological functions of hub lncRNAs in ccRCC. These hub lncRNAs were significantly abnormally expressed in ccRCC tissues and cells. Furthermore, knockdown of AC016773.2, AC024060.2, and AC026401.3 inhibited cell proliferation, migration, and invasion, suggesting that these lncRNAs play an oncogenic role in ccRCC, whereas knockdown of EMX2OS increased proliferation, migration, and invasion, suggesting that EMX2OS plays a tumor-suppressive role in ccRCC. In conclusion, our results confirm that these key lncRNAs play an important role in ccRCC.

Our study revealed an important role of necroptosis in ccRCC and established a clinically applicable NRL model. Patients with ccRCC were divided into different risk groups according to the model, and the clinical value of the model was determined through a series of correlation analyses. However, our study had some limitations. Since no large cohort containing gene expression and clinical information was found, we only used TCGA data for model construction and validation; therefore, testing the applicability of the NRL model in other datasets is necessary. In addition, our *in vitro* experiments were limited, and further *in vitro* experiments are needed to verify the mechanism of action of these NRLs in ccRCC.

## 5 Conclusions

Necroptosis is a new type of cell death that can provide new insights into tumor research. In this study, we examined the expression of necroptosis-related genes, identified prognostic NRLs, and constructed a novel NRL model. The model can not only be used as an effective index for predicting the clinical characteristics and immune features of ccRCC but can also provide a basis for guiding clinical chemotherapy and immunotherapy for ccRCC patients. In conclusion, our study found a close relationship between necroptosis and ccRCC and provided a basis for clinical prediction and treatment decision-making in ccRCC patients through a novel NRL model, which is helpful in promoting accurate individualized diagnosis and treatment.

## Data availability statement

The original contributions presented in the study are included in the article/**Supplementary Material**, further inquiries can be directed to the corresponding authors.

## Ethics statement

The studies involving human participants were reviewed and approved by First Hospital of Shanxi Medical University. The patients/participants provided their written informed consent to participate in this study.

## Author contributions

LZ, YC and TB conceived the project. LZ, YC and WH contributed to the data acquisition, analysis and manuscript writing. LZ, YC, BW, LY, DW, TB conducted the experiments and revised the manuscript. All authors read and approved the submitted manuscript. LZ, BW, DW, TB provided the funding and supervised the whole study. All authors contributed to the article and approved the submitted version.

## Supplementary material

Supplementary Figure 1 Stratified prognostic power assessment. K-M survival analysis between patients in the high- and low-risk groups in different clinical groups. Age **(A)**, stage **(B)**, grade **(C)**, M stage **(D)**, N stage **(E)**.

Supplementary Figure 2 Diagnostic value of the NRL model. **(A–E)** Correlation analysis of risk score and clinical parameters (Age, gender, T stage, N stage and M stage). **(F–H)** Differences in risk scores among patients with different clinical traits (Age, gender, and N stage).

Supplementary Figure 3 Relationship between the NRL model risk score and immune cell infiltration. **(A–L)** Correlation analysis between the risk score and the CIBERSORT immune cell infiltration level.
