# Supplementary figures and images for: A novel necroptosis-related long noncoding RNA model for predicting clinical features, immune characteristics, and therapeutic response in clear cell renal cell carcinoma
Source: Front Immunol. 2023 Aug 2;14:1230267. doi: 10.3389/fimmu.2023.1230267 (PMC10433381; doi:10.3389/fimmu.2023.1230267)

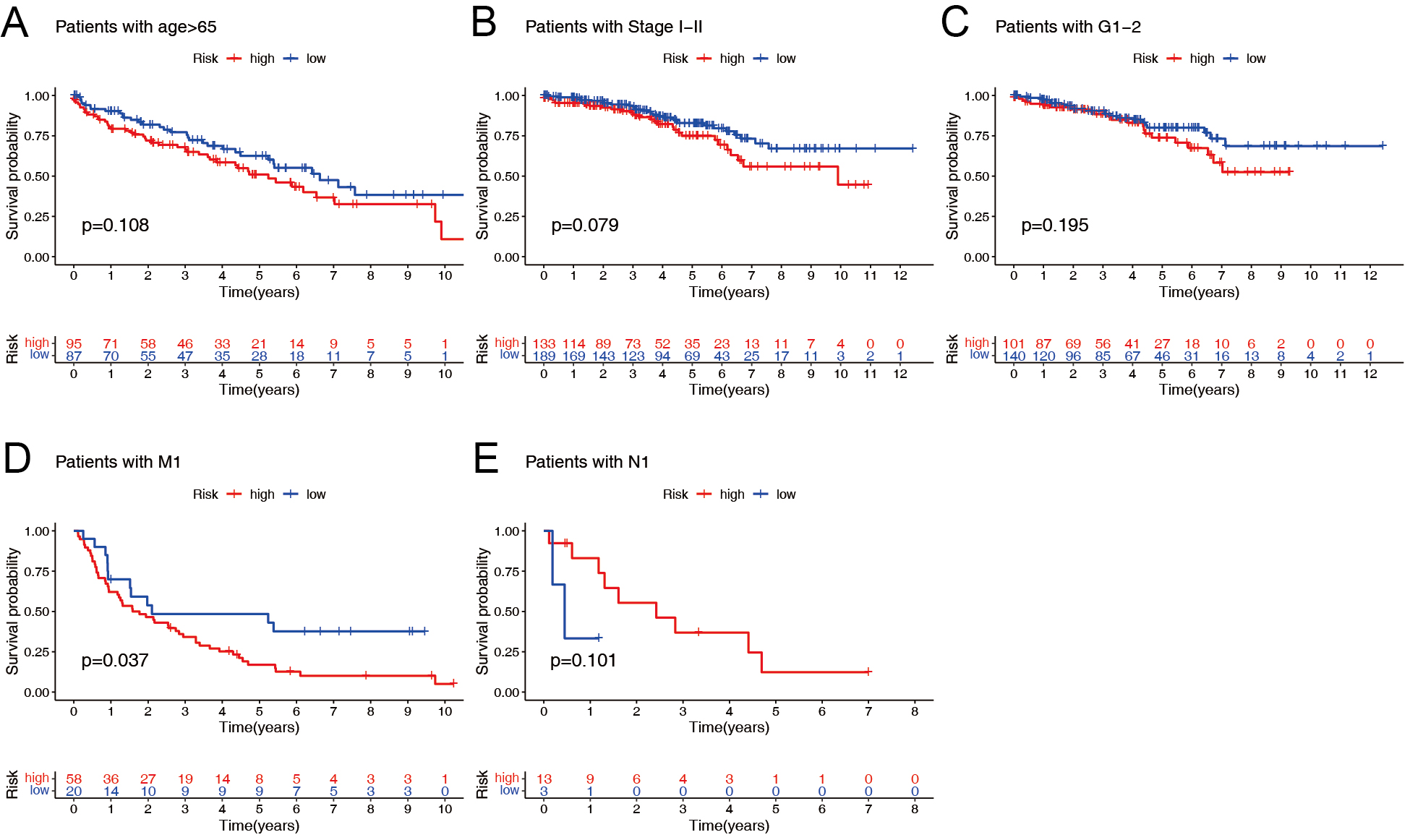

Supplement: Supplementary Figure 1 — Stratified prognostic power assessment. K-M survival analysis between patients in the high- and low-risk groups in different clinical groups. Age (A), stage (B), grade (C), M stage (D), N stage (E). [file Image_1.jpg]

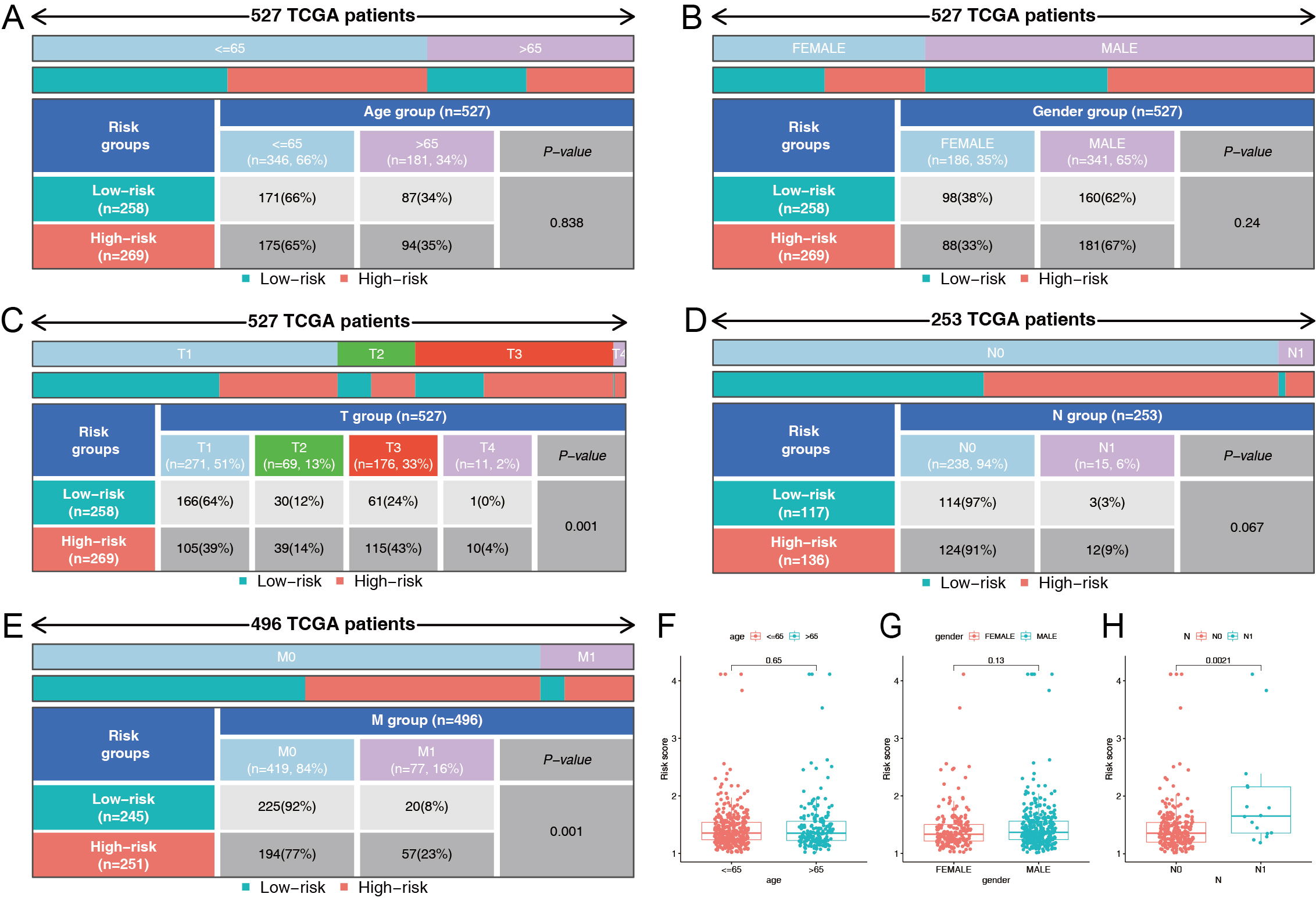

Supplement: Supplementary Figure 2 — Diagnostic value of the NRL model. (A–E) Correlation analysis of risk score and clinical parameters (Age, gender, T stage, N stage and M stage). (F–H) Differences in risk scores among patients with different clinical traits (Age, gender, and N stage). [file Image_2.jpg]

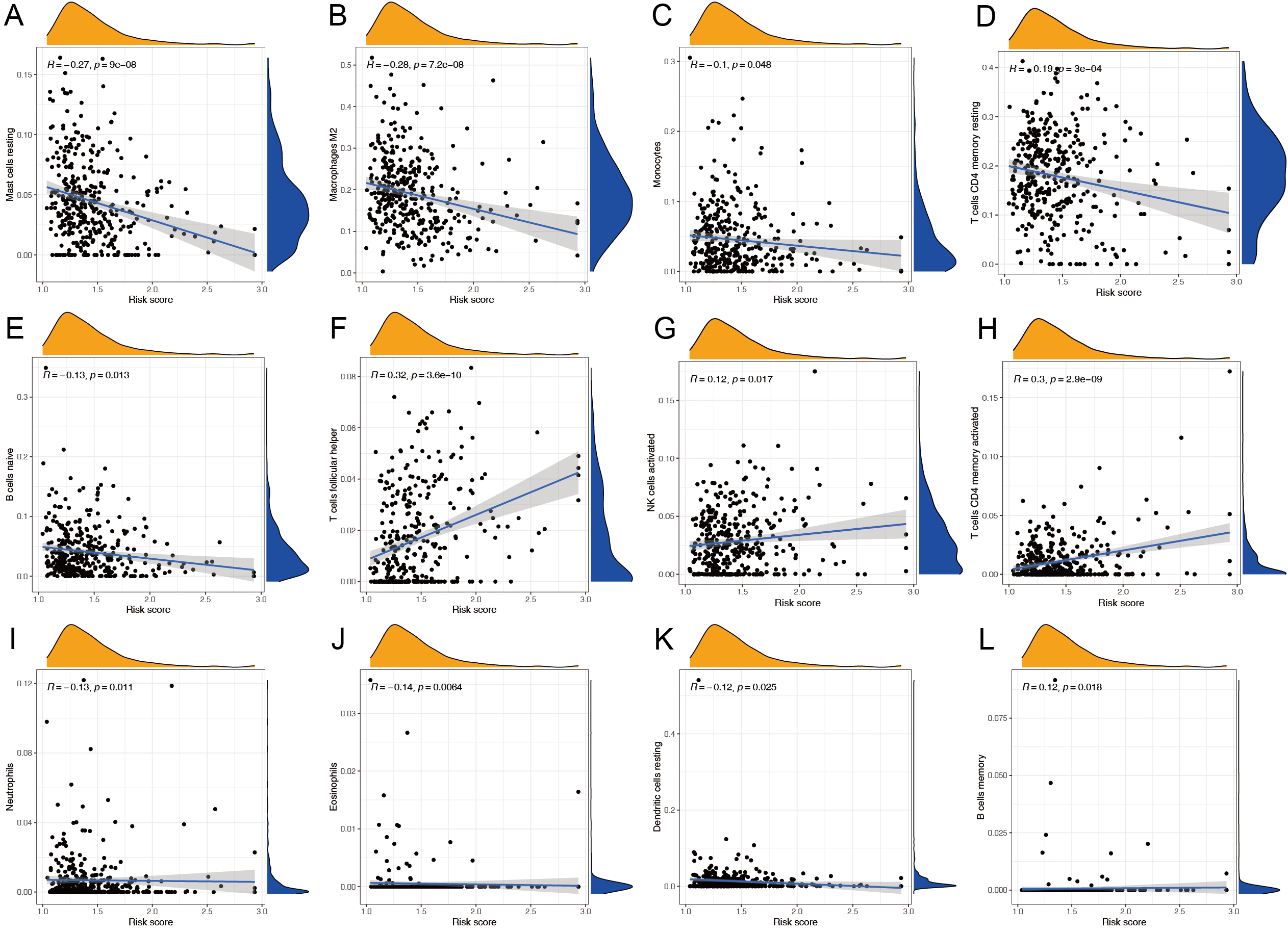

Supplement: Supplementary Figure 3 — Relationship between the NRL model risk score and immune cell infiltration. (A–L) Correlation analysis between the risk score and the CIBERSORT immune cell infiltration level. [file Image_3.jpg]
